# Le syndrome de Mirizzi: une cause rare de l’obstruction des voies biliaires: à propos d’un cas et revue de littérature

**DOI:** 10.11604/pamj.2017.27.45.12469

**Published:** 2017-05-18

**Authors:** Habib Bellamlih, Lamiae Bouimetarhan, Hassan En-nouali, Touria Amil, Naoufal Chouaib, Said Jidane, Mostafa Rafai, Ahmed Belkouch, Lahcen Belyamani

**Affiliations:** ¹Service d’imagerie médicale, Hôpital Militaire Mohamed V, Faculté de médecine et de pharmacie, Rabat, Maroc; ²Service des urgences médico-chirurgicales, Hôpital Militaire Mohamed V, Faculté de médecine et de pharmacie, Rabat, Maroc

**Keywords:** Syndrome de Mirizzi, ictère, cholangiographie rétrograde endoscopique, fistule, cholécysto-biliaire, Mirizzi’s syndrome, jaundice, endoscopic retrograde cholangiography, gallbladder, biliary fistula

## Abstract

Le syndrome de Mirizzi est une complication rare de la lithiase vésiculaire chronique, avec une incidence de 0,7% à 1,4% chez les malades cholécystéctomisés. Il est caractérisé par un ictère cholestatique en rapport avec une compression de la voie biliaire principale par un calcul enclavé dans le collet vésiculaire ou le canal cystique. La maladie peut évoluer vers l’érosion de la paroi du canal hépatique commun et par conséquent, provoquer la formation d’une fistule cholécysto-bilaire. Nous présentons ici un syndrome de Mirizzi type I en soulignant l’importance du diagnostic préopératoire, celui-ci étant rendu plus aisé par la cholangiographie rétrograde endoscopique ou par la cholangio-IRM, qui permet d’éviter des lésions iatrogènes de la voie biliaire principale. Le présent article passe en revue la littérature disponible sur les divers aspects de ce syndrome, y compris sa pathogenèse, son diagnostic et sa prise en charge.

## Abstract (en)

Mirizzi’s syndrome is a rare complication of chronic vesicular lithiasis with prevalence ranging from 0.7% to 1.4% among patients who have undergone cholecystectomy. It is characterized by cholestatic icterus associated with compression of the common bile duct due to lodged calculus in the vesicular neck or in the cystic duct. The disease can evolve toward the erosion through the common hepatic duct wall and, therefore, it can cause the formation of a gallbladder-biliary fistula. We here report a case of Mirizzi’s syndrome type I in order to highlight the role of preoperative diagnosis which is made easier by endoscopic retrograde cholangiography or by cholangio-MRI, allowing to avoid iatrogenic bile duct injuries. We conducted a review of the available literature on various aspects of this syndrome, including its pathogenesis, diagnosis and management.

## Introduction

Le syndrome de Mirizzi (SM) se définit comme une compression extrinsèque de la voie biliaire principale par un calcul enclavé dans la poche de Hartmann ou dans le canal cystique. C’est une complication rare de la lithiase vésiculaire. Dans certains cas, il peut être associé à une fistule cholécysto-biliaire secondaire à une érosion de la voie biliaire principale par le calcul enclavé. Ce syndrome peut se voir aussi dans les suites très lointaines d’une cholécystectomie [1]. Nous en rapportons un cas à la lumière duquel nous rappellerons les caractères épidémiologiques, cliniques, diagnostiques et thérapeutiques de ce syndrome.

## Patient et observation

**Figure 1:**
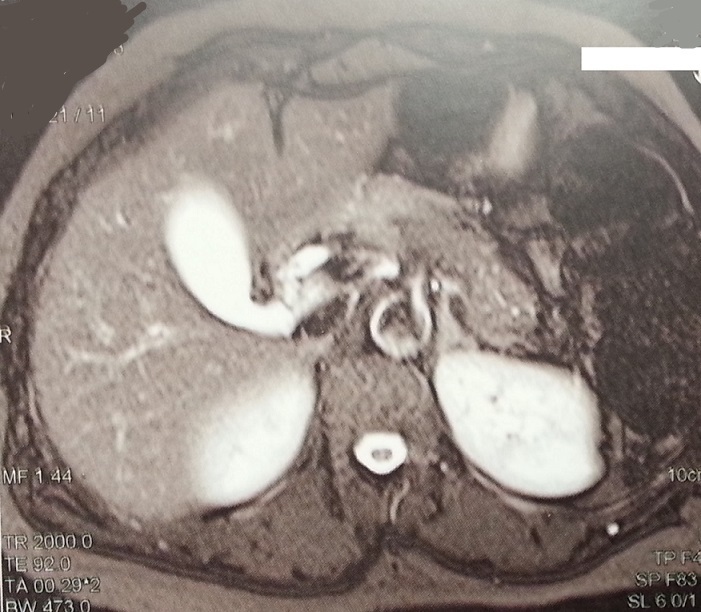
Cholangio-IRM, séquence pondérée T2 en coupe axiale, montrant une discrète dilatation des VBIH, une VB distendue en amont d’une lithiase, siégeant au niveau de l’infundibulum

**Figure 2:**
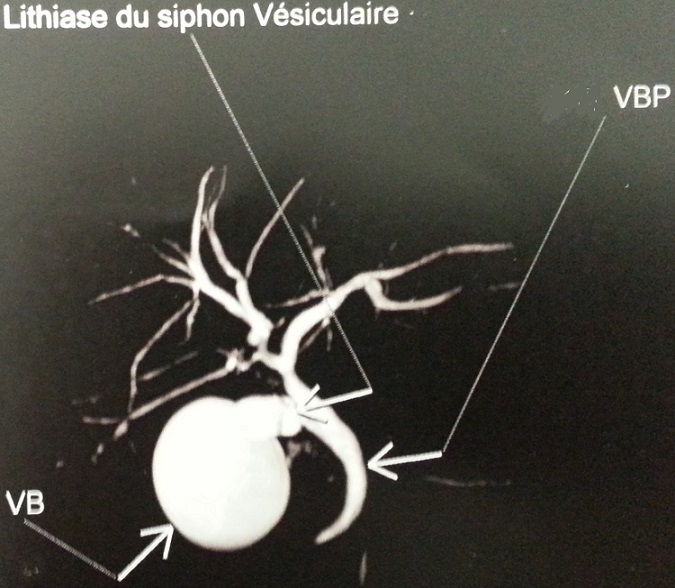
Cholangio-IRM qui montre une discrète dilatation des VBIH secondaire à un calcul enclavé au niveau du siphon de la VB qui est distendue

Mme B.L., âgée de 47 ans, sans antécédents pathologiques particuliers, a été admise au service des urgences pour douleurs abdominales diffuses plus marquées au niveau de l’hypochondre droit. L’histoire clinique remontait à 03 mois auparavant par l’installation progressive de coliques hépatiques épisodiques avec apparition une semaine avant son hospitalisation, d’une fièvre et d’un ictère d’allure cholestatique. L’examen physique révélait une patiente en bon état général avec ictère franc, fébrile à 38,4°C, une hépatomégalie à 14 cm de flèche hépatique sur la ligne mammelonnaire. Le bilan biologique retrouvait une CRP à 45 mg/l, des phosphatases alcalines à 356 UI/l, sans cytolyse hépatique. L’hémogramme montrait une hyperleucocytose à 11000/mm^3^ avec 72% de polynucléaires neutrophiles. L’échographie abdominale a mis en évidence une vésicule biliaire (VB) distendue à contenu finement échogène, à paroi épaissie régulière, avec une légère dilatation des voies biliaires intrahépatiques (VBIH), sans mise en évidence d’obstacle. Devant ce tableau clinique, une cholangiographie IRM a été réalisée (Figure 1 et Figure 2), montrant une discrète dilatation des VBIH, sans dilatation nette de la VBP (6,3mm de diamètre) et sans obstacle de type lithiasique ou tumorale sur le trajet des voies biliaires, avec une VB distendue en amont d’une lithiase, siégeant au niveau de l’infundibulum, un aspect fortement évocateur d’un syndrome de Mirizzi. La patiente a été opérée, il s’agissait d’un hydrocholécyste en amont d’un calcul du siphon vésiculaire, le canal hépatique commun était légèrement comprimé, avec un cholédoque fin et libre. Les suites opératoires sont simples avec retour à la normale du bilan biologique en 02 semaines. Un bilan biologique et une échographie de contrôle à 3 mois étaient sans particularités.

## Discussion

Le syndrome de Mirizzi a été décrit pour la première fois par KEHR en 1905 puis par RUGE en 1908 [2]. En 1948, Pablo MIRIZZI en Argentine décrit le « syndrome fonctionnel» de l’hépatique résultant uniquement de l’obstruction complète ou partielle du canal hépatique par un calcul enclavé dans le cystique avec inflammation aiguë ou chronique [3]. Actuellement, la notion de «syndrome fonctionnel» n’est plus de mise [2] et le SM se résume uniquement au syndrome de rétention biliaire par compression de la VBP par un calcul enclavé dans le cystique. Sur le plan épidémiologique, Initialement, cette entité était considérée rare pour susciter l’intérêt d’être rapporté avec seulement quelques cas isolés [2]. Actuellement des séries plus larges sur le SM sont rapportées dans la littérature [4]. L'incidence rapportée varie entre 0,05 et 4% chez les patients ayant subi une chirurgie pour lithiase vésiculaire [3]. Ces chiffres sont assez considérables vu la prévalence élevée de la lithiase vésiculaire et des cholécystectomies réalisées dans le monde entier. La SM est plus fréquent chez les femmes, probablement dû à la prépondérance des calculs biliaires chez ce groupe de patients. L'âge moyen varie entre 21-90 ans dans différentes séries [1]. L'obstruction biliaire telle qu’elle a été décrite dans le SM original résulterait de l’enclavement des calculs dans le canal cystique entraînant une inflammation et un spasme du sphincter dans le canal hépatique commun (CHC). L'entité était donc appelé «syndrome hépatique fonctionnel» [2]. Par la suite, la cause de l’obstruction s'est révélée purement mécanique, résultat d'une compression extrinsèque et d'une inflammation par le calcul dans la poche de Hartmann ou dans le canal cystique. La pathogénie a été clarifiée et les différents éléments [1] de ce syndrome ont été identifiés comme suit: canal kystique parallèle au CHC; impaction d'un calcul biliaire dans le canal cystique ou dans le col de la VB; obstruction mécanique partielle du CHC par le calcul lui-même ou par l'inflammation résultante; cholangite récurrente ou même une cirrhose biliaire au décours de l’évolution due à une obstruction partielle.

La fistule cholécysto-biliare (FCB), fistule biliaire interne entre la VB et le CHC a d'abord été décrite par Puestow en 1942 et par la suite par d'autres auteurs [1]. Différentes hypothèses ont été proposées pour expliquer sa pathogenèse. On croyait que la fistule représentait une anomalie congénitale avec absence du canal cystique. L’hypothèse la plus plausible, cependant, a été l’occlusion du canal cystique due au calcul et à l'inflammation menant à la distension de la VB et à la proximité de son mur avec celui du CHC, menant à une nécrose de la paroi commune et à la formation de fistule [1]. McSherry considère le FCB comme un stade avancé du SM, où le calcul enclavé érode le CHC formant une fistule. Sur cette hypothèse, il a classé SM en deux types: type I - Obstruction du canal biliaire par compression extrinsèque; type II - Fistule cholécysto-biliaire [5]. Csendes et al. [6] a subdivisé le FCB en fonction de l'étendue de l’érosion circonférentielle du CHC et a proposé une nouvelle classification. Les différents types selon cette classification et leur incidence relative: type I - Obstruction du CHC par le calcul enclavé dans l’infundibulum ou dans le canal cystique - 11%; type II - Fistule cholécysto-biliaire avec érosion de moins de 1/3 de la circonférence du CHC - 41%; type III - Erosion des 2/3 de la circonférence du CHC - 44%; type IV - Destruction complète de la paroi du CHC - 4%. Chaque type était considéré comme représentant la forme la plus avancée de la précédente. Cette classification semble être assez rationnelle et devrait être acceptable à tout chirurgien opérant un patient pour SM. Toutefois, l'étendue de l’érosion de la voie biliaire qui est à la base de cette classification, n'est pas identifiable par les moyens d'imagerie préopératoire, ce qui limite l’utilisation de cette classification. Mc Sherry [5] suggère une autre classification, encore répandue chez les chirurgiens et radiologues. Cliniquement, La douleur est le symptôme le plus commun chez 54 à 100% des patients suivi de l’ictère chez 24-100% des patients. La triade biliaire de Charcot est présente chez 44 à 71% des patients. La présence d'un ictère indolore peut souvent mimer une obstruction biliaire d’origine maligne. La cholécystite aiguë a été rapportée chez un tiers des patients [4]. Les présentations moins fréquentes incluent la pancréatite aiguë, la perforation de la VB et l’amaigrissement. L'hépatomégalie est commune et un carcinome mimétique de la VB peut être palpable chez 22% des patients. Rarement le patient peut être complètement asymptomatique malgré l’érosion du CHC [4]. Sur le plan biologique, on trouve une cholestase simulant celle de la lithiase vésiculaire avec un taux sérique de bilirubine allant de la normale à plus de 30 mg/dl avec une moyenne de 7-10 mg/dl. Le taux sérique des phosphatases alcalines varie de la normale à environ trois à dix fois la normale [4]. Les moyens d'imagerie sont, cependant, le pilier du diagnostic préopératoire. L'échographie est une bonne méthode de dépistage, avec une sensibilité de 23 à 46% [2]. Bien que, le plus souvent, le diagnostic suspecté par cet examen est seulement la lithiase vésiculaire [4]. Les signes échographiques spécifiques de ce syndrome sont, une VB contractée contenant des calculs, un nodule échogène ou des calculs dans le CHC, une dilatation légère à modérée des voies biliaires proximales et une voie biliaire principale de taille normale en aval des calculs ou nodules [4]. La visualisation d'une structure de type septum longitudinal dans le CHC, une apparence résultant du canal cystique dilaté en parallèle à la voie biliaire, peut également aider au diagnostic de SM dans les cas suspects. Une sensibilité (97%) et spécificité (100%) très élevées ont été rapportées dans une étude, grâce à l’échographie endocanalaire [7]. La tomodensitométrie (TDM), quand elle est réalisée, peut corroborer les résultats de l’échographie. En outre, la présence d’une cavité irrégulière adjacente au col vésiculaire contenant la protubérance du calcul, est assez caractéristique de la SM. Le duodénum et le côlon peuvent être vu à proximité immédiats de la VB représentant une fistule bilioentérique, un phénomène courant chez ces patients. La TDM a une sensibilité similaire à l’échographie, mais peut être utile pour diagnostiquer d'autres causes d’ictère obstructif telles que le cancer vésiculaire, le cholangiocarcinome ou une tumeur métastatique [4]. Malgré ces caractéristiques, le rôle de l’échographie et de la tomodensitométrie sont principalement d'exclure que de diagnostiquer un SM. La cholangiographie demeure de loin la méthode la plus fiable pour le diagnostic pré-opératoire de ce syndrome. Les résultats typiques retrouvés sur le cholangiogramme sont un défect excentrique ou excavé sur la paroi latérale de la voie biliaire au niveau du canal cystique ou du col vésiculaire. La VB peut être visualisée ou non. La voie biliaire est dilatée en amont de l’obstruction et est de calibre normal en aval [4]. Des résultats cholangiographiques similaires peuvent également être vus dans le carcinome du canal cystique ou le carcinome vésiculaire [8]. Le contour du défaut de remplissage peut être irrégulier en cas de malignité, contrairement au contour lisse du calcul dans le SM. La cholangiographie rétrograde endoscopique est préférée à la cholangiographie transhépatique ou intraveineuse percutanée car, en plus du diagnostic, elle permet l’extraction concomitante des calculs des voies biliaires présentes chez une proportion significative de ces patients [4]. Il est également possible de prévoir un drainage biliaire préopératoire chez les patients à haut risque par introduction d’un cathéter de drainage endoscopique naso-biliaire [1]. Ce dernier, aide à l'identification per opératoire du canal cystique. La CPRE n’est pas dépourvue de complications telles que le sepsis et la pancréatite, de même elle peut être limitée par un défaut de cannulation des canaux biliaires observé dans 5 % à 10 % [9]. Cependant, la Cholangiographie-IRM, grâce à son excellente résolution en contraste et à son caractère non invasif et non irradiant, occupe une place importante dans le diagnostic pré opératoire du SM. Elle permet de mettre en évidence le calcul enclavé au collet vesiculaire ou dans le canal cystique, la compression du CHC, la dilatation des canaux biliaires en amont de l’obstacle. En plus, grâce à la séquence pondérée T2, elle peut différencier une masse tumorale d’une lésion inflammatoire. La détection des petits calculs à 2mm est possible, mais les calculs enclavés ou non entourés de bile hyperintense sont parfois difficiles à détecter [10]. Le traitement du SM est chirurgical. En l'absence de diagnostic pré-opératoire définitif, une exploration chirurgicale minutieuse permet d’affirmer ou non le diagnostic dans les cas douteux. La présence d’adhérences péri vésiculaire, de VB atrophique et sessile avec ou sans fistule cholécysto-entérique, devrait suspecter un SM [4,8]. Le calcul enclavé peut simuler un carcinome du col vésiculaire, rendant la situation encore plus compliquée. Un cholangiogramme per-opératoire réalisé de début de l’acte opératoire confirme le diagnostic et contribue à la délimitation de l'anatomie biliaire [4]. Un abord antérograde de la VB est la recommandation uniforme pour les cas suspects ou confirmés [4, 8, 9]. En l'absence d'érosion des voies biliaires, c'est-à-dire un SM de type I, la cholécystectomie partielle seule est adéquate [4, 8, 10]. Après le retrait du calcul, la poche de Hartmann ou le canal cystique parallèle sont laissés derrière. Les adhérences inflammatoires entre ces structures et CHC contre indique toute dissection dans cet air, et est susceptible de provoquer des lésions des voies biliaires dans le cas inverse. En cas de FCB une approche prudente doit être envisagée. Quand l'érosion des voies biliaires est significative, des sténoses tardives ont été rapportées avec une fermeture simple de la fistule ou une réparation de bout en bout [4]. Dans la plupart des cas, le défect du CHC peut être géré en retenant une manchette de VB autour de la fistule qui est approchée, la procédure ainsi connue sous le nom de cholédochoplastie [1]. Csendes a défini des procédures bien établies pour les différents types du SM: Type I - cholécystectomie partielle; Type II - fermeture de la fistule par suture ou une cholédochoplastie; Type III - cholédochoplastie; type IV - anastomose bilioentérique. Un résultat satisfaisant avec un suivi moyen de 5,7 années est un témoignage de l'efficacité de ces procédures [1]. Dans le SM, les méthodes classiques d'extraction endoscopique échouaient, en raison de l'incapacité à capturer les calculs. Cependant, avec le développement technologique endoscopique, il est maintenant possible de traiter certains de ces patients avec succès. Dans une série de Binmoeller et al [10], 14 patients ont été sélectionnés pour un traitement endoscopique après être considéré comme non candidats à la chirurgie pour diverses raisons. En utilisant un “système scope bébé-mère”, la / les calculs ont été visualisés et fragmentés à l'aide de lithotripsie électrohydraulique (LEH) introduite par son canal fonctionnel. La durée médiane requise pour l'ensemble de la procédure était de 60 minutes. Le traitement a réussi dans tous les cas sauf un patient. Sous contrôle cholangioscopique, la LEH du calcul par voie percutanée transhépatique a également été rapportée par Cairns et al [1]. Le succès à long terme semble être constaté chez les patients de type II, qui n’ont pas de calculs vésiculaires résiduels. Angleterre et al. [10] a également montré que le traitement endoscopique est une mesure temporaire effective avant la chirurgie et peut être définitif pour les candidats non candidats à la chirurgie.

## Conclusion

Le syndrome de Mirizzi est une cause rare mais bien décrite d’ictère obstructif. Le diagnostic préopératoire du syndrome est crucial afin d’éviter les complications d’une fistule cholécysto-bilaire ou cholécystoentérique méconnue, ainsi que les lésions biliaires iatrogènes dues à la nature inflammatoire chronique du processus. Cette certitude diagnostique est essentielle en vue de définir la stratégie opératoire, laquelle est facilitée par les techniques d’imagerie telles que la CRE et la cholangiographie-IRM. L’histoire naturelle du syndrome ne s’arrête pas à la compression ou à la fistule cholécystobilaire, mais elle peut aboutir à des fistules complexes aux organes adjacents.

## Conflits d’intérêts

Les auteurs ne déclarent aucun conflit d'intérêts.
